# Supplementary material for: WordCommentsAnalyzer: A windows software tool for qualitative research
Source: F1000Res. 2018 Sep 4;7:536. Originally published 2018 May 3. [Version 2] doi: 10.12688/f1000research.14819.2 (PMC6137411; doi:10.12688/f1000research.14819.2)
Supplement: Supplementary file 3 [file f1000research-7-17614-s0002.tgz › b2a79496-4ada-47c4-9682-a99ce867836d.zip!/Eight Iranian Health Journals/Journal of Research in Rehabilitation Sciences _38.docx]

© Journal of Research in Rehabilitation Sciences . This work is licensed under CC BY-NC 4.0 (https://creativecommons.org/licenses/by-nc/4.0/).

Volume & Issue: دوره 8، شماره 3: 1391

Title: اثربخشي آموزش راهبردهاي شناختي در بهبود مهارت‌های حل مسأله رياضي در دانش‌آموزان دختر كم‌توان ذهني پايه سوم راهنمايي

http://jrrs.mui.ac.ir/index.php/jrrs/article/view/415

مقدمه: مطالعات گسترده‌ای در مورد اثربخشی آموزش راهبردهای شناختی در دانش‌آموزان با ناتوانی یادگیری انجام شده است، اما در مورد تأثیر آموزش این راهبردها در دانش‌آموزان کم‌توان ذهنی مطالعات کمی وجود دارد. هدف از اين بررسی، تعیین میزان اثر آموزش راهبردهاي شناختي در بهبود مهارت‌هاي حل مسأله رياضي در دانش‌آموزان کم‌توان ذهنی پایه سوم راهنمایی در شهرستان‌های استان تهران بود. مواد و روش‌ها: پژوهش حاضر يك مطالعه آزمايشي با طرح پيش‌آزمون- پس‌آزمون با گروه شاهد بود. 30 دانش‌آموز دختر پایه سوم راهنمایی کم‌توان ذهنی در این پژوهش شرکت داشتند که به روش خوشه‌ای چند مرحله‌ای از مدارس استثنایی شهرستان‌های تهران انتخاب شدند. این دانش‌آموزان به صورت تصادفی به دو گروه 15 نفری تقسیم شدند (یک گروه آزمایش و یک گروه شاهد). پس از توزیع تصادفي دانش‌آموزان در گروه آزمايش و شاهد، براي دانش‌آموزان گروه آزمايشي طي 15جلسه از آموزش راهبردهاي شناختي استفاده شد و به دانش‌آموزان گروه شاهد آموزش راهبردها ارایه نشد. برای ارزیابی میزان هوش‌بهر گروه‌های مورد مطالعه از آزمون هوشی Wechsler's subtests و برای ارزیابی میزان پیشرفت تحصیلی دانش‌آموزان از آزمون معلم ساخته برای بهبود مهارت‌هاي حل مسأله رياضي استفاده شد. داده‌هاي به دست آمده با استفاده از آمار توصيفي (ميانگين، انحراف معيار و جدول فراواني) و آزمون t تجزيه و تحليل شد. یافته‌ها: ميانگين نمرات آزمون حل مسأله رياضي گروه راهبرد شناختي به طور معنی‌داري بيشتر از گروه شاهد بود (0/001 > P). اين مداخله باعث افزايش ميانگين نمرات گروه آزمايش در حل مسأله رياضي نسبت به گروه شاهد شد. نتیجه گیری: يكي از حوزه‌هاي شناختي كه دانش‌آموزان كم‌توان ذهني در آن دچار اشكال هستند، حل مسأله رياضي می‌باشد. آموزش راهبرد شناختي باعث بهبود مهارت حل مسأله رياضي دانش‌آموزان كم‌توان ذهني می‌شود. با توجه به نتایج این پژوهش می‌توان عنوان کرد که اثربخشی آموزش راهبرد شناختي بر مهارت حل مسأله رياضي بيشتر از گروه شاهد بوده است.

Title: تأثير تمرين کوتاه‌مدت همراه با ويبراسيون کل بدن بر فعاليت الکتروميوگرافي عضلات سولئوس و گاستروکنميوس در زنان جوان سالم

http://jrrs.mui.ac.ir/index.php/jrrs/article/view/559

مقدمه: اثرات ويبراسيون کل بدن (Whole body vibration یا WBV) بر پاسخ الکترومايوگرافي عضلات بررسي شده است، اما پاسخ عضلات کند و تند انقباض پس از اعمال WBV مشخص نيست. هدف از اين مطالعه، بررسي اثر تمرين کوتاه‌مدت همراه با WBV بر فعاليت الکتريکي عضلات سولئوس و گاستروکنميوس به عنوان عضلات کند و تند انقباض بود. مواد و روش‌ها: دوازده زن سالم جوان غير ورزشکار (سن، 2/71 ± 25/66 سال؛ قد، 5/6 ± 161 سانتي‌متر؛ وزن، 5/42 ± 66/5 کيلوگرم) به صورت تصادفي در دو گروه درمان‌نما (Sham) (6 = n) و WBV (6 = n) قرار گرفتند. قبل و بعد از 12 جلسه تمرين، دامنه فعاليت الکترومايوگرافي (Root mean square یا RMS) حين حداکثر انقباض ايزومتريک ارادي عضلات سولئوس و گاستروکنميوس ثبت شد. تمرين شامل حفظ وضعيت سمي اسکوات ايزومتريک بود که در گروه درمان‌نما روي دستگاه WBV خاموش و در گروه WBV روي دستگاه WBV روشن (فرکانس ويبراسيون 30 هرتز و دامنه قله به قله جابجايي صفحه ويبراسيون 3 ميلي‌متر) انجام گرفت. یافته‌ها: فعاليت الکتريکي عضلات سولئوس و گاستروکنميوس قبل از شروع تمرينات در دو گروه يکسان بود (0/05 < P). دوازده جلسه تمرين تغيير معني‌دار آماري در RMS عضلات سولئوس و گاستروکنميوس هيچ يک از گروه‌ها ايجاد نکرد (0/05 < P). همچنين، با وجود تغيير رفتار و افزايش پاسخ RMS عضله گاستروکنميوس خارجي از گروه درمان‌نما به گروه WBV، تفاوت معني‌داري در مقدار تغيير RMS عضله مذکور بين دو گروه مشاهده نشد (0/05 < P).  مقدار تغيير RMS عضله سولئوس نيز تفاوت معني‌داري بين دو گروه نداشت (0/05 < P). نتیجه گیری: با اين که افزايش فعاليت الکتريکي عضلات سولئوس و گاستروکنميوس بعد از تمرين WBV از نظر آماري به سطح معني‌دار نرسيد، اما بررسي الگوي پاسخ عضلات بين گروه درمان‌نما و گروه WBV، مبين اثر مثبت WBV بر عضله گاستروکنميوس به عنوان عضله تند انقباض است.

Title: ارتباط بين حافظه فعال و رواني کلامي در سالمندان

http://jrrs.mui.ac.ir/index.php/jrrs/article/view/308

مقدمه: حافظه فعال و رواني کلامي از اولين عملکردهاي شناختي هستند که در فرايند سالمندي افت پيدا مي‌کنند. هدف از اين مطالعه بررسي، ارتباط بين اين دو عملکرد شناختي در سالمندان بود. مواد و روش‌ها: در اين مطالعه 150 نفر از سالمندان ساکن در استان قم، به صورت تصادفي خوشه‌اي انتخاب شدند و به آزمون‌هاي رواني كلامي (معنايي و آوايي) و آزمون فراخناي اعداد (مستقيم و معکوس) پاسخ دادند. جهت بررسي ارتباط بين متغيرهاي مطالعه از آزمون همبستگي Pearson دو طرفه استفاده شد. یافته‌ها: بين رواني کلامي آوايي و بخش مستقيم آزمون فراخناي اعداد همبستگي معني‌داري وجود داشت (0/05 < P، 0/286 = r). همچنين بين بخش معکوس آزمون فراخناي اعداد و آزمون رواني کلامي آوايي نيز همبستگي معني‌داري نشان داده شد (0/01 < P، 0/355 = r). رواني کلامي معنايي نيز همبستگي معني‌داري با فراخناي اعداد مستقيم (0/01 < P، 0/599 = r) و معکوس (0/01 < p، 0/488 = r) نشان داد. نتیجه گیری: ارتباط بين رواني کلامي آوايي و معنايي را مي‌توان به دليل زيرساخت‌هاي تشريحي مشترک اين دو کارکرد شناختي (قطعه پيش پيشاني و به طور اختصاصي بخش طرفي خارجي آن) دانست. بر اين اساس مي‌توان آزمون رواني کلامي را به عنوان پيش‌گوي حافظه فعال دانست.

Title: ارتباط مشکلات تغذیه‌ای و عقب‌ماندگی ذهنی

http://jrrs.mui.ac.ir/index.php/jrrs/article/view/387

مقدمه: غذا خوردن یک کارکرد انطباقی ضروری بوده است و هر گونه نقص در مهارت‌های تغذیه‌ای نه تنها بر کیفیت زندگی افراد عقب‌مانده ذهنی، بلكه بر رشد آموزشی، اجتماعی و ذهنی آن‌ها نيز تأثير مي‌گذارد. پژوهش حاضر با هدف، بررسی رابطه بین مشکلات تغذیه‌ای و عقب‌ماندگی ذهنی انجام شده است. مواد و روش‌ها: در این پژوهش توصیفی₋ تحلیلی، 114 كودك عقب‌مانده ذهني با روش نمونه‌گیری تصادفی ساده انتخاب شدند. به منظور گردآوری داده‌ها پرسش‌نامه متغيرهاي دموگرافيك، آزمون هوش Stanford–Binet و نیز پرسش‌نامه مشکلات تغذیه‌ای مورد استفاده قرار گرفت. به منظور تجزیه و تحلیل داده‌ها از نرم‌افزار 12SPSS استفاده شد. یافته‌ها: از افراد مورد پژوهش 63/2 درصد مذکر و میانگین سن نمونه‌های مورد پژوهش 8/53 سال بود. يافته‌ها نشان داد که بین مشکلات تغذیه‌ای و شدت عقب‌ماندگی ذهنی ارتباط مثبت و معنی‌داری وجود دارد (0/001 > P). نتیجه‌گیری: مشکلات ناشی از اختلالات تغذیه‌ای کودکان عقب‌مانده ذهنی در هر یک از حیطه‌های آن شیوع بالایی دارد و با افزایش شدت عقب‌ماندگی ذهنی، این مشکلات افزایش می‌یابد.

Title: بررسی روایی و اعتبار نسخه فارسی آزمون واژه‌یابی ویرایش دوم در كودكان 6-4 ساله طبيعي مهد كودك‌هاي شهر اصفهان: فرم پیش دبستانی

http://jrrs.mui.ac.ir/index.php/jrrs/article/view/520

مقدمه: مشکلات واژه‌یابی در میان کودکان دارای اختلالات زبانی و یادگیری وجود دارد. از رایج‌ترین راه‌های ارزیابی واژه‌یابی، ارزیابی رسمی است که آزمون واژه‌یابی ویرایش دوم یکی از آن‌ها می‌باشد. در این مطالعه، به بررسی روایی و اعتبار آزمون واژه‌یابی ویرایش دوم در کودکان 6-4 ساله مهد کودک‌های شهر اصفهان پرداخته شده است. مواد و روش‌ها: مطالعه حاضر توصیفی- مقطعی بود. سؤالات آزمون پس از ترجمه و انجام تغییرات لازم بر روی 157 کودک 6-4 ساله، به صورت تصادفی و با کسب اجازه از والدین ایشان اجرا شد. تحلیل سؤالات با استفاده از نظریه سؤال- پاسخ و با وارد نمودن الگوی پاسخ‌ها به صورت صفر و یک در نرم‌افزار 6BILOG-MG انجام شد. اعتبار آزمون با استفاده از روش Cronbach's alpha به دست آمد و همچنین شاخص‌های آماری فرم پیش ابتدایی با استفاده از نرم‌افزار 13SPSS محاسبه شد. یافته‌ها: میانگین نمرات واژه‌یابی در سنین 4، 5 و 6 ساله به ترتیب برابر با 23/75، 27/14 و 28/55 به دست آمد که نشان دهنده افزایش تدریجی میانگین با افزایش سن می‌باشد (0/05 = α، 0/01 > P). اعتبار آزمون نیز برابر با 0/79 بود. همچنین جهت تأمین روایی آزمون، درجه دشواری و قدرت تمییز سؤالات آزمون بر اساس دو نظریه تستی کلاسیک و سؤال- پاسخ محاسبه شد. نتیجه‌گیری: میانگین نمرات واژه‌یابی فرم پیش ابتدایی کودکان فارسی زبان با میانگین نمرات مطالعه German همخوانی داشت. از آن جایی که قدرت تمییز سؤالات آزمون برای کودکان فارسی زبان نسبت به کودکان انگلیسی زبان بالاتر است، به نظر می‌رسد بهتر از نمونه اصلی توانسته است بین افراد دارای توانایی‌های مختلف تمییز ایجاد کند. اعتبار آزمون برابر با اعتبار آزمون اصلی به دست آمد. بدين ترتيب یک آزمون روا و معتبر جهت ارزیابی واژه‌یابی کودکان فارسی زبان به دست آمد.

Title: مقایسه فرکانس پایه در کودکان دارای شنوایی عادی و کودکان دچار درجات مختلف افت شنوایی

http://jrrs.mui.ac.ir/index.php/jrrs/article/view/484

مقدمه: شنوایی یکی از حواس مهم در کنترل حلقه بسته و از عوامل تأثیر‌گذار بر روی صوت می‌باشد؛ چرا که پس‌نورد (Feedback) لازم را برای کنترل صوت فراهم می‌کند. در افراد با افت شنوایی به دلیل عدم برخورداری از چنین پسنوردی، توانایی کنترل حرکتی عضلات مربوط به ساز و کارهای آواسازی و تولید گفتار تحت تأثیر قرار می‌گیرد. در نتیجه نمی‌توانند فرکانس پایه (Fundamental frequency یا F0) خود را تنظیم کنند که منجر به کاهش وضوح گفتار در آن‌ها می‌شود. هدف پژوهش حاضر، مقایسه پارامتر آکوستیکی F0 در کودکان با درجات مختلف افت شنوایی با همتاهای عادی بود. مواد و روش‌ها: صدای 28 کودک دارای افت شنوایی (متوسط، متوسط تا شدید، شدید و عمیق) و 28 کودک دارای شنوایی عادی در گروه سنی 7 تا 9 سال مورد بررسی قرار گرفت. شرکت کنندگان تک زبانه، فارسی زبان بودند. از آزمودنی‌ها خواسته شد که واکه /a/ را سه بار بکشند. میانگین F0 با استفاده از نرم‌افزار Praat نسخه 5.3.13 محاسبه شد. جهت تعیین وجود تفاوت بین میانگین F0 در گروه‌ها از آزمون One way ANOVA و برای تعیین تفاوت معنی‌دار در گروه‌ها آزمون Post hoc DUNNET استفاده شد. یافته‌ها: بیش‌ترین و کمترین میزان F0 به ترتیب در گروه افت شنوایی عمیق و گروه عادی بود. نتایج آزمون ANOVA نشان داد که میانگین F0 در گروه‌ها متفاوت است (0/025 = P) و این تفاوت به لحاظ آماری بین F0 کودکان دارای افت شنوایی عمیق و گروه شاهد بود (0/005 = P).  نتیجه گیری: میانگین F0 با افزایش افت شنوایی، افزایش می‌یابد. این حالت ممکن است به عنوان مکانیزمی برای جبران نبود پس‌نورد شنیداری باشد. آن چه در این پژوهش به دست آمد، نشان می‌دهد که با تشدید افت شنوایی میانگین F0 افزایش می‌یابد، ولی تعیین مرز بین طبیعی و مرضی بودن صدای نمونه‌های مورد مطالعه نیازمند پژوهش دیگری است.

Title: تأثیر یک دوره برنامه تمرین استقامتی در آب بر تعادل ایستا، پویا و قدرت اندام تحتانی مردان ارتشی سالمند سالم

http://jrrs.mui.ac.ir/index.php/jrrs/article/view/471

مقدمه: سالمندان در معرض ضعف تعادل، افتادن و در پی آن آسیب، کاهش استقلال و مرگ زودرس قرار دارند. هدف از انجام پژوهش حاضر، بررسی تأثیر يك دوره برنامه تمرین استقامتی در آب بر تعادل ایستا، پویا و قدرت عضلاني اندام تحتاني مردان سالمند بود. مواد و روش‌ها: مطالعه نیمه تجربی حاضر، دارای یک طرح پیش‌آزمون- پس‌آزمون دو گروهه (شاهد، تمرین در آب) بود. آزمودنی‌های تحقیق را 19 مرد سالمند (60 تا 70 ساله) عضو کانون بازنشستگان ارتش شهرستان کرج تشکیل دادند. گروه تمرین در آب، یک برنامه تمرین استقامتي (هوازي و عضلاني) شش هفته‌اي (دو جلسه در هفته) را در استخر انجام دادند. در طول دوره، گروه شاهد در برنامه تمرینی خاصی شرکت نداشتند. تعادل ایستا، پویا و قدرت عضلانی اندام تحتانی تمام آزمودنی‌ها به ترتیب با استفاده از آزمون‌های زمان ایستادن روی یک پا با چشمان باز و بسته، زمان برخاستن و رفتن و برخاستن از صندلی سالمندان قبل و بعد از دوره تمرین، در شرایطی مشابه اندازه‌گیری شد. از آزمون t همبسته به منظور مقایسه نتایج پیش و پس‌آزمون و آزمون Independent t جهت مقایسه بین دو گروه استفاده شد. یافته‌ها: تعادل ایستا با چشمان باز (0/002 = P) و بسته (0/001 = P)، تعادل پویا (0/001 = P) و قدرت عضلانی (0/001 = P) در گروه تمرین در آب بهبود معنی‌داری داشته است، لکن تغییرات معنی‌داری در گروه شاهد مشاهده نشد (0/05 < P). به علاوه در تمامی آزمون‌ها تفاوت معنی‌داری بین نتایج پیش‌آزمون گروه تمرین در آب و شاهد وجود نداشت (0/05 < P)، اما بین نتایج میزان پیشرفت دو گروه تفاوت معنی‌داری وجود داشت (0/05 > P). نتیجه‌گیری: با توجه یافته‌های تحقیق، به نظر می‌رسد تمرينات استقامتی هوازي و عضلاني در آب را می‌توان برای بهبود تعادل مردان سالمند سالم توصیه نمود.

Title: بررسي رابطه بين مهارت‌هاي اجتماعي و رشد زبان دريافتي در کودکان 4 تا 6 ساله فارسي زبان

http://jrrs.mui.ac.ir/index.php/jrrs/article/view/349

مقدمه: زبان و مهارت اجتماعي دو مشخصه نوع بشر هستند که در زندگي انسان نقش بسيار مهمي ايفا مي‌کنند. اکتساب گفتار و زبان يکي از مهم‌ترين مؤلفه‌هاي رشد است که کاملاً وابسته به محيط اجتماعي شکل مي‌گيرد. در اين بين، زبان بياني و دريافتي دو عنصر کليدي رشد زبان محسوب مي‌شوند که نقش مهي را در رشد و گسترش کفايت اجتماعي کودک بازي مي‌کنند. هدف از اين پژوهش، بررسي وجود ارتباط بين رشد زبان دريافتي و ابعاد مهارت‌هاي اجتماعي در کودکان 4 تا 6 ساله فارسي زبان بود. مواد و روش‌ها: در اين پژوهش، 123کودک 4 تا 6 سال (76 پسر و 47 دختر) که معيارهاي ورود به مطالعه را داشتند، از طريق تصادفي چند مرحله‌اي از بين همه مهدکودک‌هاي شيراز (144 مهدکودک)، انتخاب شدند. در ابتدا از اين کودکان آزمون رشد زبان و مقياس نظام درجه‌بندي مهارت‌هاي اجتماعي که شامل دو فرم معلم و والدين است، گرفته شد. داده‌هاي حاصله با استفاده از روش ضريب همبستگي Pearson، توسط نرم‌افزار 16SPSS و در سطح معنی‌داري 0/05 مورد تجزيه و تحليل قرار گرفت. یافته‌ها: ضريب همبستگي Pearson ارتباط مثبت و معنی‌داري را بين تحول زبان دريافتي و ابعاد مهارت‌هاي اجتماعي فرم معلم نشان ‌داد (0/01 > P)، ولي همبستگي معنی‌دار بين تحول زبان دريافتي و ابعاد مهارت‌هاي اجتماعي فرم والدين وجود نداشت (0/01 < P). همچنين، بين نظرات معلم و والدين همبستگي معنی‌داري وجود نداشت (0/01 < P). نتیجه گیری: يافته‌هاي تحقيق حاضر، رابطه مثبت معنی‌داري را بين تحول زبان دريافتي و ابعاد مهارت‌هاي اجتماعي آشکار ساخت که اين ارتباط تنها از ديدگاه معلمان معنی‌دار است. بر اين اساس، بايد به شرايط متفاوت محيط خانه و محيط آموزشي در امر تعليم و تربيت مهارت اجتماعي به کودکان توجه کرد و در اين راستا سعي کرد که آموزش‌هاي لازم را در هر دو محيط براي آن‌ها فراهم نمود؛ چرا که رشد اين مهارت‌ها مي‌تواند به رشد زبان دريافتي کودکان کمک کند.

Title: بررسي تأثير آموزش Handling به مراقبين در منزل، بر تکامل حرکتي ظريف در کودکان فلج مغزي 72-6 ماهه

http://jrrs.mui.ac.ir/index.php/jrrs/article/view/473

مقدمه: هدف اين مطالعه، تعيين تأثير آموزش مراقبت‌هاي ويژه روزمره زندگي به والدين، در منزل بر تکامل حرکتي ظريف در کودکان فلج مغزي 72-6 ماهه بود. مواد و روش‌ها: در اين مطالعه تجربي، 40 کودک 6 تا 72 ماهه مبتلا به فلج مغزي از بين مراجعه کنندگان به کلينيک‌هاي توان‌بخشي تهران، مطابق معيارهاي تحقيق انتخاب شدند و در دو گروه شاهد و مداخله قرار گرفتند. محقق در منزل کودکان گروه مداخله حضور می‌یافت و همراه با تطبیق منزل، آموزش‌های مربوط به مراقبت‌های ویژه روزمره زندگی را طی 1 جلسه 4 ساعته به مراقبین کودک ارایه می‌داد. ابزار جمع‌آوري داده‌ها شامل فرم مشخصات دموگرافيک، GMFCS (Gross motor function classification system) و PEABODY (Peabody developmental motor scales and activity cards test) بود. داده‌ها در 3 نوبت: قبل از دوره مداخله، 1/5 ماه و 3 ماه بعد از دوره مداخله  ثبت می‌شد. یافته‌ها: تکامل حرکتي ظريف در هر دو گروه، 1/5 و 3 ماه بعد از دوره مداخله نسبت به قبل تفاوت معنی‌داري داشت (0/001 > P). ميانگين پيشرفت نمرات تکامل حرکتي ظريف 3 ماه بعد از دوره مداخله، در گروه مداخله بهتر از گروه شاهد بود (0/043 = P) و همچنین تفاوت میانگین پیشرفت نمرات تکامل حرکتی ظریف 1/5 ماه بعد از دوره مداخله، در دو گروه معنی‌دار نبود (0/19 = P). ميانگين پيشرفت استفاده از دست، دستکاري، هماهنگي چشم و دست در گروه مداخله بيشتر از گروه شاهد بود، اما تفاوت معنی‌دار نبود. در حالی که ميانگين پيشرفت "در دست گرفتن اجسام" در گروه مداخله به طور معنی‌داري بيشتر از گروه شاهد بود (0/036 = P). نتيجه‌گيري: نتایج این مطالعه این گونه نشان می‌دهد که آموزش Handling به مراقبين در منزل، در طولاني مدت (3 ماه) باعث بهبود تکامل حرکتي ظريف کودکان فلج مغزي 6 تا 72 ماهه مي‌شود.

Title: تأثير آموزش مهارت‌هاي زندگي بر ميزان مهارت‌هاي اجتماعي دانش‌آموزان کم‌‌شنواي فراگير

http://jrrs.mui.ac.ir/index.php/jrrs/article/view/431

مقدمه: مطالعات گسترده‌اي در مورد اثربخشي آموزش مهارت‌هاي زندگي بر رفتار دانش‌آموزان انجام شده است، اما در مورد اثربخشي آموزش مهارت‌هاي زندگي بر رفتار دانش‌آموزان داراي معلوليت پژوهش‌هاي کمي وجود دارد. با توجه به اين که نياز اين گروه از دانش‌آموزان به آموزش مهارت‌ها بيشتر از سايرين است، لزوم برنامه‌ريزي و اجرا در اين خصوص اهميت ويژه‌اي دارد. هدف از اين پژوهش، تعيين اثربخشي آموزش مهارت‌هاي زندگي بر افزايش ميزان مهارت‌هاي اجتماعي دانش‌آموزان پسر کم‌‌شنواي مقطع راهنمايي مدارس فراگير بود. مواد و روش‌ها: طرح پژوهش از نوع مطالعات شبه آزمايشي با پيش‌آزمون و پس‌آزمون همراه با دو گروه آزمايش و شاهد بود. از ميان 38 دانش‌آموز پسر داراي مشکل شنوايي که در رده‌هاي اول تا سوم راهنمايي در مدارس فراگير (تلفيقي) شهر اراک در سال تحصيلي 91-1390 مشغول به تحصيل بودند، به صورت تصادفي 30 نفر از آن‌ها انتخاب و به دو گروه 15 نفري آزمايشي و شاهد تقسيم شدند. گروه آزمايشي هفته‌اي دو بار و طي 16 جلسه دو ساعته، مهارت‌هاي زندگي را آموزش ديدند. مهارت‌هاي اجتماعي هر دو گروه قبل و بعد از آموزش با استفاده از مقياس درجه‌بندي مهارت‌هاي اجتماعي (Social skills rating scale) ارزيابي شد. براي تجزيه و تحليل داده‌هاي گرد‌آوري شده از پيش‌آزمون و پس‌آزمون، آمار توصيفي و آزمون‌هاي آماري Independent t و ANCOVA استفاده شد. یافته‌ها: گروه آزمايش نسبت به گروه شاهد عملکرد بهتري در پس‌آزمون مهارت‌هاي اجتماعي داشت (0/001 > P). نتیجه گیری: آموزش مهارت‌هاي زندگي، مهارت‌هاي اجتماعي، همکاري، مهارت ابراز وجود و خويشتن‌داري (مهار خود) را به گونه‌اي معنی‌دار افزايش مي‌دهد.

Title: بررسي تأثير يک دوره تمرينات پيلاتس بر بهبود عملکردهاي حرکتي و شناختي مرتبط با افتادن در زنان سالمند

http://jrrs.mui.ac.ir/index.php/jrrs/article/view/522

مقدمه: رسيدن به سن سالمندي از عمده‌ترين پيشرفت‌هاي بشر است، ولي کاهش عملکرد حرکتي به ويژه تعادل و اختلالات شناختي از عوامل کليدي افتادن در سالمندان است که با عواقب و عوارض جسماني، روان‌شناختي و اجتماعي زيادي همراه است. بنابراين هدف از تحقيق حاضر، بررسي تأثير برنامه تمريني پيلاتس بر بهبود عملکردهاي حرکتي و شناختي مرتبط با افتادن در زنان سالمند بود. مواد و روش‌ها: اين پژوهش يک مطالعه نيمه تجربي بود. 30 زن سالمند با ميانگين سن: 5/9 ± 70/23 سال، وزن:10/53 ± 61/67 کیلوگرم و قد: 7/88 ± 150/43 سانتی‌متر که در سال1390 به مرکز توان‌بخشي جامع سالمندان فرهيختگان (شهرکرد) مراجعه کردند، به صورت در دسترس و هدفمند انتخاب شدند و به طور تصادفي در دو گروه تجربي و شاهد قرار گرفتند. به منظور ارزيابي كاركرد حرکتي از آزمون دسترسي عملكردي و آزمون 10 متر راه رفتن و براي ارزيابي كاركرد شناختي از آزمون حافظه باليني Wechsler (فرم الف) استفاده شد. سپس گروه تجربي به مدت 12 هفته برنامه تمرين درماني پيلاتس را دريافت کردند. تجزيه و تحليل آماري با استفاده از آزمون ANCOVA در سطح معني‌داري 0/05 > P با کمک نرم‌افزار 16SPSS انجام شد. يافته‌ها: بين دو گروه تجربي و شاهد تفاوت معني‌داري در آزمون دسترسي عملكردي (0/037 = P، 4/789 = F) و آزمون 10 متر راه رفتن در سرعت معمولي (0/002 = P، 12/345 = F) و سرعت حداکثر (0/002 = P، 11/575 = F) مشاهده شد. همچنين در آزمون حافظه باليني Wechsler تفاوت معني‌داري بين گروه تمرين پيلاتس و شاهد مشاهده گردید (0/001 = P، 13/127 = F). نتیجه‌گیری: نتايج تحقيق حاضر نشان داد که برنامه تمريني پيلاتس مي‌تواند بر بهبود عملکرد حرکتي به ويژه تعادل و عوامل شناختي مرتبط با افتادن در زنان سالمند مؤثر باشد. بنابراين به نظر مي‌رسد ورزش پيلاتس روش تمريني سودمندي جهت حفظ عملکرد حرکتي و کاهش سرعت کارکردهاي ذهني سالمندان باشد و خطر افتادن و هزينه‌هاي درماني وابسته به آن را کاهش دهد.

Title: ارزیابی تأثیر برنامه آموزش مراقبت‌های ویژه بر دانش مادران از نحوه مراقبت صحیح از کودکان فلج مغزی

http://jrrs.mui.ac.ir/index.php/jrrs/article/view/560

مقدمه: با توجه به مشكلات متعدد كودكان فلج مغزي در انجام فعاليت‌هاي روزمره زندگي و مشكلات مراقبين اين كودكان در نگهداري از آن‌ها، ارایه روش‌هاي مؤثر به خانواده براي مراقبت از كودك در زمينه‌هاي غذا خوردن، حمام كردن، تحرك در محيط منزل و غيره می‌تواند براي به دست آوردن استقلال بيشتر در زندگي روزانه مؤثر باشد. نقش کاردرمانی در این حیطه، آموزش و مشاوره به مراقبین در زمینه وضعیت‌دهی و در رابطه با مداخلاتی است که عملکرد کاری کودک را تسهیل و باعث کاهش فشارهای جسمانی و روانی بر مراقبین گردد. هدف از این پژوهش، بررسی میزان آگاهی مراقبین از شیوه‌های صحیح مراقبت و تعیین اثربخشی برنامه آموزشی بر آن بود. مواد و روش‌ها: در مطالعه کارآزمایی بالینی حاضر، 60 مادر کودک فلج‌ مغزی 12-4 ساله مراجعه‌ کننده به مراکز توان‌بخشی شهر تهران در سال 91-1390، به روش در دسترس انتخاب شدند. نمونه‌ها به روش تصادفی به دو گروه مداخله و شاهد تقسیم گردید. میزان آگاهی مادران با استفاده از پرسشنامه ميزان آگاهي از مراقبت‌هاي ويژه از كودكان فلج مغزي و شدت فلج مغزی با سیستم طبقه‌بندی عملکرد حرکتی درشت مورد بررسی قرار گرفت. اطلاعات جمع‌آوری شده با شاخص‌های مرکزی و پراکندگی و با استفاده از آزمون آماری Independent t و واریانس یک طرفه تحلیل گردید. یافته‌ها: میانگین سن مادران در گروه شاهد 32/8 سال و در گروه مداخله 35/3 سال بود. سنجش ميزان آگاهي بر اساس آزمون، نشان داد که 85/2 درصد از مادران دارای آگاهي ضعیف و متوسط بودند میزان آگاهی خوداظهاری (0/002 = P) و آگاهی بر اساس آزمون (0/001 > P) مادران کودکان فلج مغزی گروه مداخله، از شیوه‌های صحیح مراقبت پس از دریافت برنامه آموزشی، در مقایسه با گروه شاهد تغییر قابل ملاحظه‌ای نمود و افزایش نشان داد. نتیجه‌گیری: با توجه به نتایج فوق می‌توان نتیجه‌گیری کرد که دانش مادران پیرامون مراقبت صحیح از کودک فلج مغزی کافی نیست و برنامه آموزشی حاضر بر ارتقای دانش مادران تأثیر مثبتی داشته است.

Title: مقایسه اثر تمرینات ثباتی و مکنزی بر روی درد، ناتوانی و اندازه عضله مولتی فیدوس کمری در زنان مبتلا به کمردرد غیر اختصاصی مزمن

http://jrrs.mui.ac.ir/index.php/jrrs/article/view/413

مقدمه: كمردرد مزمن منجر به علايم باليني متعدد مانند مهار رفلكسي عضلات مولتي فيدوس و آتروفي آن، اختلال عملكردي و آتروفي عضلات پارااسپينال ناحیه کمر و ناتوانی می‌گردد كه اين آتروفي شانس عود كمردرد را افزايش مي‌دهد. بنابراین در اين مطالعه اثر تمرینات ثباتی و مکنزی بر روی درد، ناتوانی و اندازه عضلات مولتی فیدوس کمری در بیماران مبتلا به کمردرد مزمن غیر اختصاصی مقایسه گردید. مواد و روش‌ها: اين مطالعه با طراحي كارآزمايي باليني با شرکت 60 خانم غیر ورزشکار مبتلا به کمردرد مزمن غیر اختصاصی انجام شد. بیماران به طور تصادفی در دو گروه تمرینات ثباتی و مکنزی قرار گرفتند. میزان درد، ناتوانی و سطح مقطع عضله مولتي فيدوس قبل، پس از 6 هفته مداخله شامل تمرینات ورزشی و همین طور پس از 4 هفته پیگیری به ترتیب با استفاده از مقیاس دیداری درد، پرسشنامه ناتوانی Oswestry و اولتراسونوگرافی اندازه‌گیری شد. از آزمون Independent t براي تجزيه و تحليل داده‌ها استفاده گردید. مقادير 0/05 < P به عنوان سطح معني‌داري در نظر گرفته شد. يافته‌ها: تمرینات ثباتی و مکنزی باعث کاهش معنی‌دار درد و ناتوانی نسبت به دوره قبل از تمرین شدند (0/0001 < P). همچنین تمرینات ثباتی منجر به افزایش معنی‌دار اندازه عضله در سطح مهره‌ای پنجم گردید (0/0001 < P)، در حالی که تمرینات مکنزی تغییر معنی‌داری روی اندازه عضله نداشت (0/05 > P). نتیجه‌گیری: تمرینات ثباتی و مکنزی سبب کاهش درد و ناتوانی در بیماران مبتلا به کمردرد مزمن غیر اختصاصی می‌شوند. به علاوه تمرينات ثباتي باعث افزايش اندازه عضله مولتي فيدوس در سطح مهره پنجم گردید. با توجه به اين اثر مضاعف به نظر مي‌رسد که استفاده از تمرينات ثباتي بتواند در جلوگيري از عود مجدد كمردرد مؤثر باشد.

Title: بررسی الگوی زمانی خیشومی شدگی در گفتار کودکان فارسی زبان 4 تا 12 ساله با و بدون شکاف کام

http://jrrs.mui.ac.ir/index.php/jrrs/article/view/657

مقدمه: پرخیشومی از رایج‌ترین اختلالات تشدید در کودکان دارای شکاف کام است. الگوهای خیشومی شدگی در زبان‌های مختلف، متفاوت می‌باشد. زمان‌بندی ضعیف حرکات کامی- حلقی در ارتباط با سایر اندام‌های تولیدی منجر به پرخیشومی می‌شود. اگر چه ارزيابي ادراكي، استانداردي طلايي براي ارزيابي پرخیشومی می‌باشد، ولی دارای مشکلاتی است که از اعتبار آن می‌کاهد. تجزیه و تحلیل‌های آکوستیکی می‌تواند مکمل ارزیابی‌های ادراکی باشد. هدف این مطالعه، بررسی تفاوت الگوی زمانی گفتار در کودکان دارای شکاف کام و طبیعی فارسی زبان می‌باشد و این که کدام بافت آوایی بیشترین تفاوت را بین دو گروه می‌گذارد. مواد و روش‌ها: امواج آکوستیکی و طیف نگاشت حاصل از نمونه صدای 14 کودک دارای شکاف کام و 14 کودک طبیعی 4 تا 12 ساله فارسی زبان که همزمان از دهان و بینی ضبط شده بودند، با استفاده از نرم‌افزار Praat مورد بررسی قرار گرفت و دیرش کل خیشومی شدگی در سه بافت pamap، pimip، pumup محاسبه گردید. داده‌ها در سطح معنی‌داری 95 درصد مورد تجزیه و تحلیل قرار گرفت (05/0 > P). یافته‌ها: کودکان دارای شکاف کام دیرش کل خیشومی شدگی طولانی‌تری نسبت به کودکان طبیعی داشتند. تفاوت دیرش کل خیشومی شدگی در دو گروه و همچنین در میان سه بافت آوایی معنی‌دار بود (001/0 > P). نتیجه‌گیری: دیرش خیشومی شدگی طولانی‌تر در گروه دارای شکاف کام نسبت به گروه طبیعی نشان داد که کودکان دارای شکاف کام الگوهای زمانی گفتاری انحراف یافته یا باتأخیر نسبت به کودکان طبیعی دارند. استفاده از ارزیابی آکوستیکی ویژگی‌های زمانی گفتار جهت بررسی کمّی ویژگی‌های تشدید در کودکان دارای شکاف کام و تمییز از کودکان طبیعی مفید می‌باشد.

Title: ارتباط ترکیب بدنی با ناهنجاری‌های لوردوز و کیفوز در بانوان

http://jrrs.mui.ac.ir/index.php/jrrs/article/view/506

مقدمه: هدف از انجام این پژوهش، مطالعه رابطه ترکیب بدنی (شاخص توده بدنی، نسبت چربی بدن، نسبت محیط کمر به باسن) با ناهنجاری‌های لوردوز و کیفوز در بانوان متقاضی تحصیل در رشته تربیت بدنی دانشگاه پیام‌نور بود. مواد و روش‌ها: جامعه پژوهش را 600 نفر از بانوان متقاضی ورود به مقطع کارشناسی رشته تربیت بدنی دانشگاه پیام‌نور که نمره قبولی در آزمون تئوری فراگیر را کسب کرده بودند، تشکیل دادند. از این تعداد 233 نفر از بانوان به صورت تصادفی با میانگین سنی 6/093 ± 27/11 سال، قد 8/533 ± 161/09 سانتی‌متر و وزن 9/34 ± 58/9 کیلوگرم انتخاب شدند. این پژوهش از نوع کاربردی و به روش توصیفی- همبستگی انجام شد. برای هر یک از نمونه‌ها اندازه‌گیری آنتروپومتری (قد، وزن و سن) به عمل آمد. ضخامت چربی زیر پوستی (سه سر بازو، چهار سر ران و فوق خاصره) با استفاده از دستگاه کالیپر به مدت 3 ثانیه و حداقل با سه تکرار از سمت راست بدن آزمودنی‌ها اندازه‌گیری شد. درصد چربی بدن با استفاده از فرمول Jackson و Pollock محاسبه گردید و میزان انحرافات ستون مهره‌ها (کیفوز و لوردوز) با دستگاه اسپینال موس اندازه‌گیری گردید. برای تجزیه و تحلیل آماری از نرم‌افزار کامپیوتری 12SPSS و روش آماری ضریب همبستگی Pearson در سطح معنی‌داری 0/05 استفاده شد. یافته‌ها: چنان چه از نتایج بر می‌آید، شاخص توده بدن با ناهنجاری لوردوز رابطه معنی‌داری داشت (0/04 = P، -0/144 = R)، در حالی که درصد چربی بدن، شاخص دور کمر و شاخص نسبت دور کمر به دور باسن با ناهنجاری لوردوز رابطه معنی‌داری نداشت (-0/037 = R، 0/58 = P). همچنین شاخص توده بدن و درصد چربی بدن با ناهنجاری کیفوز رابطه معنی‌داری نداشت (0/099 = R، 0/134 = P). به علاوه شاخص دور کمر و شاخص نسبت دور کمر به دور باسن با ناهنجاری کیفوز رابطه معنی‌داری نداشت (0/01- = R، 0/99 = P). نتیجه‌گیری: به طور کلی نتایج این پژوهش نشان داد که WHR (Waist to hip ratio)، WC (Waist circumference) و درصد چربی بدن با ناهنجاری‌های لوردوز و کیفوز رابطه معنی‌داری ندارد.BMI  (Body mass index) با ناهنجاری لوردوز رابطه معنی‌دار داشت، در حالی که با ناهنجاری کیفوز رابطه معنی‌داری نداشت.

Title: اثربخشي روش مداخله‌اي شن‌بازي درماني بر رشد ادراک حرکتي- بينایي کودکان ناتوان ذهني آموزش‌پذير

http://jrrs.mui.ac.ir/index.php/jrrs/article/view/255

مقدمه: کودکان احتياج به بازي دارند. بازي، تفکر و شيوه اظهار وجود آن‌ها است. ازجمله بازي‌هاي مورد علاقه کودکان که شاید موجب رشد آن‌ها خواهد شد، شن‌بازي است. هدف پژوهش حاضر، بررسي اثربخشي روش مداخله‌اي شن‌بازي درماني (Sandplay therapy) بر رشد ادراک حرکتي- بينایي (Visuo motor perception) کودکان ناتوان ذهني آموزش‌پذير (Educable children) بود. مواد و روش‌ها: روش تحقیق تجربی (طرح پیش‌آزمون و پس‌آزمون با گروه شاهد) بود. نمونه‌هاي اين پژوهش شامل 32 دانش‌آموز با ناتواني ذهني آموزش‌پذير بودند. ابتدا از ميان شش ناحيه آموزش‌پذير شهر اصفهان يک ناحيه به طور تصادفي و از ميان اين ناحيه يک مدرسه دو نوبته (دخترانه/ پسرانه) به طور تصادفي انتخاب گردید. بدين ترتيب تعداد 32 نفر از ميان دانش‌آموزان اين دو مدرسه از پايه‌هاي تحصيلي پيش‌دبستاني، اول، دوم و سوم به طور تصادفی انتخاب شدند. سپس اين تعداد نمونه به طور تصادفي در دو گروه 16 نفري مورد و شاهد شامل 8 دختر و 8 پسر قرار داده شدند. ابزار پژوهش عبارت از آزمون رشد شناختي- تشخيصي Kay بود. سپس کودکان ناتوان ذهني آموزش‌پذير گروه مورد طي 10 جلسه 45 دقيقه‌اي شن‌بازي درماني را در مدرسه دريافت کردند. یافته‌ها: روش مداخله‌اي شن‌بازي درماني بر رشد ادراک حرکتي- بينایي اين کودکان مؤثر بود (0/001 > P). ضمن اين که بين دو گروه دختر و پسر در نمرات پس‌آزمون رشد ادراک حرکتي- بينایي بعد از مداخله شن‌بازي تفاوت معنی‌داري مشاهده نشد. نتیجه گیری: نتايج اين پژوهش نشان داد که روش مداخله‌اي شن‌بازي درماني قادر است رشد ادراک حرکتي- بينایي کودکان ناتوان ذهني آموزش‌پذير را افزايش دهد، بنابراین استفاده از این روش به عنوان روشی در بهبود بخشی از مشکلات مربوط به رشد ادراک حرکتی- بینایی این کودکان پیشنهاد می‌شود.

Title: مقایسه حداکثر نیروهای عمودی عکس‌العمل زمین و نرخ بارگذاری در حرکت فرود تک پای مردان دارای زانوی پرانتزی و نرمال

http://jrrs.mui.ac.ir/index.php/jrrs/article/view/564

مقدمه: نرخ بارگذاری بالا با آرتروز ارتباط دارد و زانوی پرانتزی زمینه‌ساز بروز آرتروز ‌می‌‌‌باشد، درک این موضوع که وجود این ناهنجاری تا چه میزان متغیرهای بیومکانیکی را در حین فرود دستخوش تغییر می‌سازد، سبب می‌شود تا در جهت پیش‌گیری از آرتروز در افراد دارای ناهنجاری زانوی پرانتزی گام مهمی را برداریم. لذا هدف مطالعه حاضر، مقایسه‌ حداکثر نیروهای عمودی عکس‌العمل زمین و نرخ بارگذاری در حرکت فرود تک پا در مردان دارای زانوی پرانتزی و نرمال بود. مواد و روش‌ها: 40 نفر دانشجوی مرد سالم رشته تربیت بدنی و علوم ورزشی، شامل 21 نفر دارای زانوی پرانتزی و 19 نفر دارای زانوی نرمال در این تحقیق شرکت کردند. ناهنجاری زانوي پرانتزي با استفاده از كوليس اندازه‌گیری و ثبت شد. آزمودنی‌ها حرکت فرود تک پا را از ارتفاع 30 سانتی‌متر روی صفحه نیرو انجام دادند. به منظور مقایسه پارامترها بین دو گروه از روش آماری MANOVA استفاده شد. یافته‌ها: تفاوت معنی‌داری در حداکثر نیروی عمودی عکس‌العمل زمین در هنگام برخورد پنجه نشان داده نشد (0/486 = P)، اما تفاوت حداکثر نیروی عمودی عکس‌العمل زمین هنگام برخورد پاشنه (0/002 = P) و میزان‌ بار (0/048 = P) بین دو گروه معنی‌دار بود. نتیجه‌گیری: تغییرات اندک در زاویه مفصل زانو در صفحه فرونتال بر فعالیت عضلانی، گشتاورها و نیروهای مفصلی در مفاصل زانو و مچ پا تأثیر می‌گذارد .ممکن است یک دلیل بالاتر بودن خطر آسیب دیدگی و ابتلا به آرتروز در افراد دارای زانوی پرانتزی نسبت به افراد نرمال، نیروهای عکس‌العمل زمین و نرخ بارگذاری بالای اعمال شده به اندام تحتانی آن‌ها در حین فرود آمدن است و توصیه می‌شود برای کاهش خطر آسیب دیدگی و ابتلا به آرتروز، بر تمرینات اصلاحی و عمل بیومکانیکی اصلاح یافته تمرکز شود.

Title: اثربخشي آموزش مهارت‌های اجتماعی بر هوش هیجانی معلولین جسمی- حرکتی

http://jrrs.mui.ac.ir/index.php/jrrs/article/view/469

مقدمه: در اين پژوهش تأثیر آموزش مهارت‌های اجتماعی بر هوش هیجانی معلولین جسمی- حرکتی شهر مشهد مورد بررسي واقع شد. مواد و روش‌ها: روش پژوهش نیمه آزمایشی (با طرح پیش‌آزمون- پس‌آزمون با گروه شاهد) بود. جامعه آماری شامل کلیه معلولین جسمی- حرکتی (108 نفر) مجتمع آموزشی نیکوکاری توان‌یابان مشهد بود. از بین آن‌ها پس از غربال‌گری اولیه تعداد 30 نفر که از لحاظ مهارت‌های اجتماعی و سطح هوش هیجانی دارای بیش‌ترین نارسایی بودند، انتخاب شدند و با استفاده از شیوه گمارش تصادفی در دو گروه آزمایش و شاهد قرار گرفتند. سپس آموزش مهارت‌های اجتماعی در طی 15 جلسه به گروه آزمایش ارایه شد. جهت جمع‌آوری داده‌های پژوهش از پرسش‌نامه مهارت‌های اجتماعی Regio و پرسش‌نامه هوش هیجانی Bar–On استفاده گردید. تحلیل داده‌های پژوهش با استفاده از آزمون ANCOVA چند متغیری انجام شد. یافته‌ها: گروه آزمایش در مقایسه با گروه شاهد تفاوت معنی‌داري در مؤلفه‌هاي روابط درون فردی {0/01 = P، 13/61 = (22، 1)F}، روابط بین فردی {0/01 = P، 24/02 = (22، 1)F}، سازگاری {0/01 = P، 20/47 = (22، 1)F}، کنترل استرس {0/01 = P، 8/69 = (22، 1)F}، خلق عمومی {0/01 > P، 13/55 = (22، 1)F} و سطح هوش هیجانی کلی {0/02 = P، 22/16 = (22، 1)F} داشت. آموزش مهارت‌های اجتماعی بیش‌ترین اثربخشی را بر مؤلفه‌های روابط بین فردی، روابط درون فردی، خلق عمومی و سازگاری و کم‌ترین اثربخشی را بر مؤلفه کنترل استرس داشته است. نتیجه گیری: با توجه به مؤلفه‌های مهارت‌های اجتماعی، آموزش این مهارت‌ها منجر به افزایش سطح کلی هوش هیجانی معلولین جسمی- حرکتی گردید. ارتقای سطح هوش هیجانی بهبود وضعیت سلامت روانی- اجتماعی معلولین جسمی- حرکتی را به همراه دارد.

Title: مقايسه ناهنجاري‌هاي وضعیتی منتخب ستون فقرات ووشوکاران حرفه‌اي و آماتور با افراد غير ورزشکار

http://jrrs.mui.ac.ir/index.php/jrrs/article/view/466

مقدمه: اجراى الگوهاى حركتى و مهارتى هنگام تمرين و مسابقه می‌تواند موجب سازگاري‌هاى منفى در ساختار دستگاه اسكلتى شود. اين تحقيق با هدف بررسي، ناهنجاري‌هاي کيفوز، لوردوز و سر به جلو در ورزشکاران ووشوکار در دو سطح حرفه‌اي و آماتور و مقايسه آن با افراد غير ورزشکار انجام شد. مواد و روش‌ها: نمونه آماري اين تحقيق شامل 50 ورزشکار حرفه‌اي ووشوکار، 50 ورزشکار آماتور ووشوکار و 50 نفر غير ورزشکار بود. براي اندازه‌گيري کيفوز و لوردوز از خط‌کش منعطف استفاده شد. به منظور سنجش ميزان زاويه سر از گونيامتري که بدين منظور ساخته شده بود، استفاده شد. به منظور تجزيه و تحليل داده‌ها از آزمون ANOVA يک سويه و آزمون تعقيبي Tukey استفاده شد. یافته‌ها: کيفوز، لوردوز و سر به جلو در هر سه گروه داراي تفاوت معنی‌دار بود (به ترتيب 22/08 = F و 0/01 > P، 1/8 = F و 0/01 > P، 15/8 = F و 0/01 > P). در ورزشکاران حرفه‌اي با 13 ± 49/8 درجه کيفوز، 9 ± 51/2 درجه لوردوز، 7/2 ± 35/6 درجه سر به جلو بيش‌ترين ميزان اختلال پاسچرال مشاهده شد. نتیجه گیری: با توجه به اين که ناهنجاري‌هاي کيفوز، لوردوز و سر به جلوی ورزشکاران حرفه‌اي شديدتر از افراد غيرحرفه‌اي بود، طراحى و برنامه‌ريزي مناسب تمرينات تخصصي ويژه اين رشته ورزشي و توجه به حفظ پاسچر صحيح در حين تمرينات ضروري مي‌باشد.

Title: مروري بر برخي مطالعات مدل‌هاي Finite element زانو

http://jrrs.mui.ac.ir/index.php/jrrs/article/view/442

مقدمه: زانو يکي از مهم‌ترين و پرکاربردترين مفصل‌هاي بدن انسان مي‌باشد که در معرض آرتروز و بيماري‌ها قرار دارد. استفاده از مدل‌هاي بيومکانيکي و تحليل Finite element، درک صحيحي از نيروهاي ماهيچه‌اي، تنش غضروف و پيش‌بيني آسيب‌هاي زانو در وضعيت‌ها و شرايط مختلف بارگذاري ارایه خواهد نمود. در هنگام رانندگي با ماشين‌آلات زراعي به دليل نامناسب بودن طراحي و عدم برخورداري از ارگونومي مناسب، اعمال نيروي بيش از حد مجاز از طرف راننده به پدال باعث بارگذاري نامناسب مفصل زانو و در نتيجه آسيب ديدن غضروف‌ مفصلي و ايجاد آرتروز زانو مي‌شود. هدف از اين مطالعه، مروري بر نتايج حاصل از تحليل Finite element تحت سينتيک‌هاي مختلف و تحت شرايط فعاليت اپراتورهاي ماشين‌آلات کشاورزي بود. مواد و روش‌ها: بررسي بيومکانيک کل مفصل زانو و مدل‌سازي Finite element سه بعدي اين مفصل با استفاده از روش‌هاي عکس‌برداري و آناتومي هندسه، مي‌تواند سينماتيک و مکانيک اتصال و تماس در اين مفصل را مورد ارزيابي قرار دهد. همچنین با بررسي مدل استقرار و شرايط مختلف بارگذاري‌، می‌توان نيروهاي ايجاد شده را تجزيه و تحليل نمود. مدل‌‌هاي بيومکانيکي زانو اغلب شامل ساختار استخواني، لايه‌هاي غضروفي مجزا، منيسک‌ها و ليگامنت‌‌هاي اصلي مي‌باشد. یافته‌ها: مطالعه تحقيقات صورت پذيرفته روي مفصل زانو در سينتيک‌هاي مختلف، به خصوص موقعيت‌هاي مشابه فشردن پدال در ماشين‌آلات کشاورزي بيانگر اين نتيجه بود که به دليل اعمال نيروي زياد به خصوص در اکستنشن کامل زانو، مشابه فشردن پدال‌ها، سينتيک‌هاي خاص مي‌تواند در کاهش تنش‌هاي به وجود آمده تا زير حد تحمل زانو و عملکرد بهتر مفصل، مؤثر واقع شود. نتیجه‌گیری: ضرورت انجام مطالعه مروري، بر روي مفصل زانو، بررسي وضعيت‌هاي مختلف زانو و شرايط تحمل بار و چگونگي سينماتيک، رفتار و عملکرد تحت سينتيک مختلف زانو توسط روش‌هاي Finite element و بيومکانيک مي‌باشد. به ويژه در فعاليت با ماشين‌آلات کشاورزي که فرد با شرايط خاص قرارگيري، بارهاي شديد و اغلب در شرايط نامناسب ارگونوميکي روبرو مي‌باشد.
